# Use of the Capability, Opportunity, Motivation‐Behaviour Model and Theoretical Domains Framework to Understand Barriers and Enablers of Research Capacity and Culture for Speech and Language Therapy Staff

**DOI:** 10.1111/1460-6984.70116

**Published:** 2025-09-10

**Authors:** Katie Dooley Cawley, Helen Stringer

**Affiliations:** ^1^ National Institute for Health and Care Research, UK; ^2^ Cumbria, Northumberland, Tyne and Wear NHS Foundation Trust, Newcastle upon Tyne, UK; ^3^ Newcastle University Newcastle upon Tyne UK

**Keywords:** Research Capacity and Culture, speech and language therapy

## Abstract

**Background:**

Research Capacity and Culture (RCC) is important for research engagement. Little is known of what speech and language therapy staff perceives to be the barriers or enablers to this at individual, team and organisational levels.

**Aims:**

To identify the barriers and enablers to RCC among speech and language therapy staff, using behaviour change theory as a framework, and to explore their self‐reported level of research engagement.

**Methods:**

Participants completed an online survey through purposive sampling. The survey and results were analysed following the Theoretical Domains Framework (TDF) and Capability, Opportunity and Motivation (COM‐B) model, informed by the RCC Tool. Quantitative data were analysed using descriptive statistics. Percentage responses for ‘Yes’, ‘No’ and ‘Don't Know’ were categorised as barriers, enablers and don't know. Total percentage scores were classified as weak (0%–33.33%), moderate (33.34%–66.66%) or strong (66.67%–100%). Free text responses were analysed using NVivo (v12.0) and a structured categorisation matrix of barrier and enabler. Labelled emotions were the unit of analysis. Finally, participants selected a category reflecting their level of research engagement.

**Outcomes and Results:**

Fifty‐seven (response rate 73.08%) speech and language therapy staff members from an NHS Trust participated. Barriers and enablers were represented across eight domains of the TDF. At the individual level, knowledge and skill for activities linked to research‐related professional standards from the HCPC were strong or moderate enablers, except one. More advanced research activities were rated as strong or moderate level barriers. For motivation, participants' beliefs about the benefit to clinical practice and desire to engage in more research activity (91.23% and 71.93%) were strong enablers. At the team and organisational level, time was a moderate strength barrier. Overall, there was poor knowledge of the availability of support and supervision. For environmental context and resources, library access was a strong enabler (98.25%); all other factors were weak enablers. For the level of research engagement, 52.63% were ‘Research Conscious’, 24.56% ‘Research Participative’, 21.05% ‘Research Active’ and 1.75% unknown.

**Conclusions and Implications:**

Barriers and enablers to RCC were identified at all levels of study. Participants demonstrated motivation to engage in research and beliefs in its positive impact on practice. Barriers included a lack of knowledge and skills for more advanced research activities, time, resources, funding and information about the support or opportunities available. Findings provide insight into RCC for speech and language therapy as a profession.

**WHAT THIS PAPER ADDS:**

*What is already known on this subject*

- The Research Capacity and Culture (RCC) of Allied Health Professionals within the United Kingdom has previously been explored using the RCC Tool. However, no study to date has focussed specifically on the perspective of Speech and Language Therapists (SLTs) and Speech and Language Therapy Assistants (SLTAs) as a distinct professional group. The COM‐B Model and Theoretical Domains Framework (TDF) have been widely applied in evidence‐based healthcare but have not yet been used to examine RCC.

*What this paper adds to existing knowledge*

- This study provides novel insight into the perspectives of speech and language therapy staff working in a UK mental health and disability trust regarding their RCC. By applying a behaviour change lens (COM‐B and TDF), the study identifies specific strategies to overcome barriers and enhance enablers to research engagement.

*What are the potential or actual clinical implications of this work?*

- This study highlights the value of using behaviour change theory and frameworks to explore and analyse RCC. While findings are context‐specific, they contribute to a broader understanding of RCC within the speech and language therapy profession and may inform targeted approaches to strengthen research engagement across similar settings.

## 1 Introduction

Speech and language therapists (SLTs) are required to engage with research to support the delivery of evidence‐based healthcare (Health Care Professions Council 2024). This obligation also extends to speech and language therapy assistants (SLTAs) employed in speech and language therapy services. Research and innovation are critical for improving patient care, with evidence showing that research‐active healthcare providers are associated with better patient experience, enhanced patient safety and improved service performance (Jonker et al. 2020; NHS England 2019; Jonker and Fisher 2018). Increased research engagement also contributes to professional skill development, job satisfaction and the implementation of evidence into practice (Pager et al. 2012; Lizarondo et al. 2011; Newington et al. 2021). Speech and language therapy staff with direct patient contact are ideally placed to identify and deliver research tailored to the needs of the population they serve (Pager et al. 2012).

Research is embedded in national policy and recognised as a core component of health and social care (Health Research Authority 2021; NHS England 2019). Organisations are directed to support research activity and ensure that practice is underpinned by evidence (Health Education England 2022). For SLTs in the United Kingdom, there is a legal requirement to engage with research‐related activities, set out within Standard 11 of the Health and Care Professions Council (HCPC) standards of proficiency (SoP) (Health Care Professions Council 2024). Despite this, SLTs and other Allied Health Professions (AHP) are considered less research active and underrepresented in clinical academic (CA) leadership compared to their medical colleagues (Wenke and Mickan 2016).

To address this, Health Education England published a research and innovation strategy for AHPs aimed at strengthening research capacity and capability across the workforce (Health Education England 2022). Research capacity building (RCB) is defined as ‘enhancing the abilities of individuals, organisations and systems to undertake and disseminate high quality research efficiently and effectively’ (Department of International Development 2010, 28). The concept is a complex and multi‐level process that encompasses individuals, teams, organisations and networks (Cooke 2005; Condell and Begley 2007; Cooke et al. 2018). Alongside this, the concept of research culture is relevant, referring to the environment in which research occurs. It is shaped by how research is valued and supported at individual, team and organisational levels and can be represented through available resources, incentives and leadership (Wagner et al. 2005).

Several models and frameworks exist to facilitate RCB in the AHP workforce, including the Individual Participant Framework (IPF) (Whitworth et al. 2012), the Research Capacity and Culture Tool (RCCT) (Holden et al. 2012) and the Royal College of Speech and Language Therapists (RCSLT) Research Practitioners Framework (Royal College of Speech and Language Therapy 2022a, 2022b). The IPF helps individuals to reflect on their level of engagement in the research process. Users move from and between ‘Research Conscious’, ‘Research Participative’ and ‘Research Active’ on a continuum, with status changing over time, dependent on role. The framework is designed with the understanding that most users will fall into the ‘Research Conscious’ category. If completed by a staff group, results can serve as a useful marker to understand users’ perception of research engagement to compare over time. The RCCT, validated for AHPs, enables quantitative measurement of Research Capacity and Culture (RCC) across individual, team and organisational levels, with strong internal consistency in all three domains (Holden et al. 2012). It has been used in several studies to evaluate RCC (e.g., Comer et al. 2022; Cordrey et al. 2022; Lee et al. 2020; Dickens et al. 2024).

Alongside these tools, the RCSLT has developed the RCSLT Research Practitioners Framework, which provides a profession‐specific roadmap for developing research skills across varying levels of experience and responsibility (Royal College of Speech and Language Therapy 2022a, 2022b). It complements other national and organisational initiatives and can serve as a guide for embedding research activity and culture into routine practice.

**FIGURE 1:**
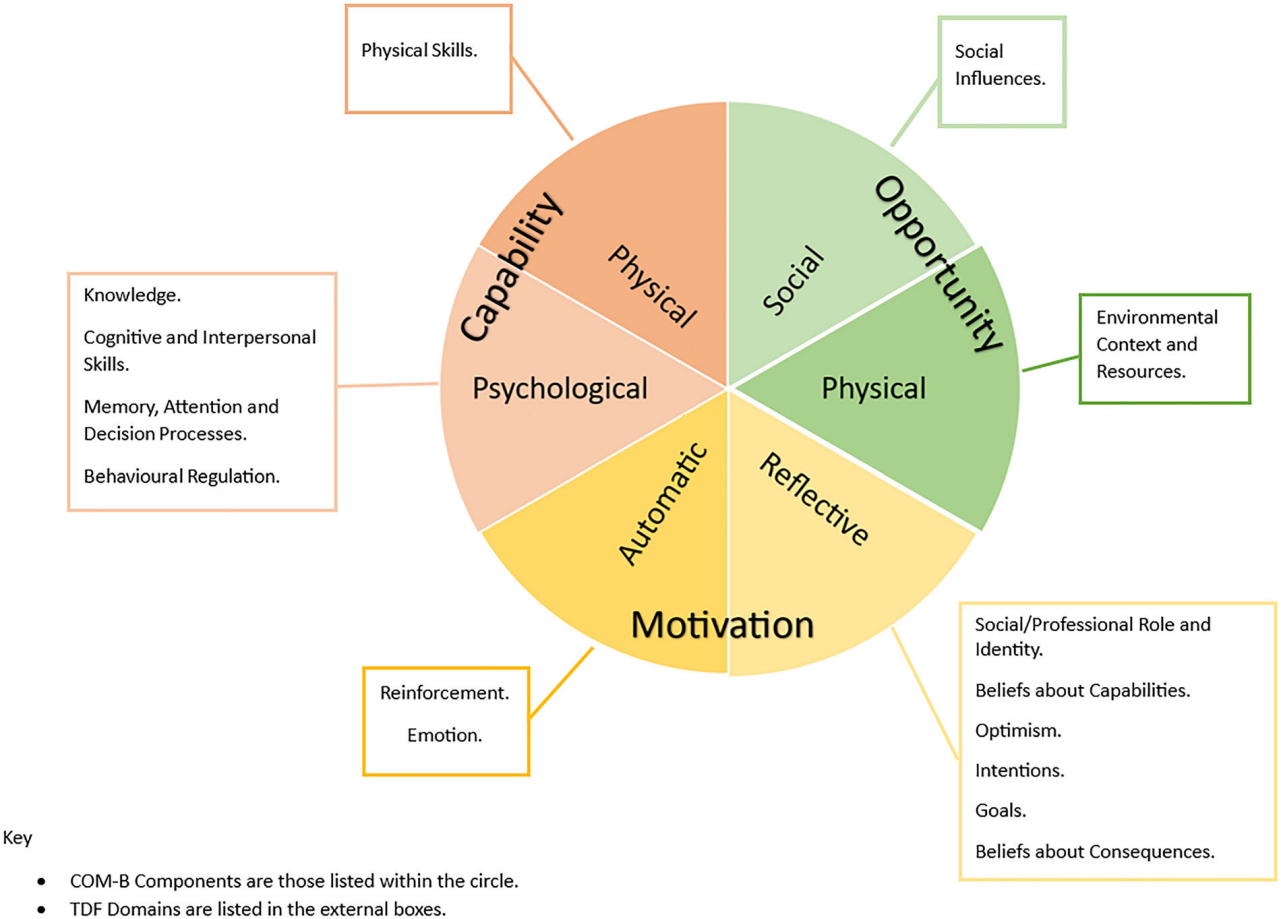
COM‐B components linked to the domains of the TDF.

While tools such as the IPF, RCCT and the Research Practitioners Framework provide valuable structure and insight, they do not explicitly incorporate psychological constructs that underpin behaviour change. Since RCB is a complex intervention, the selection and design of successful implementation strategies may benefit from grounding in behavioural theory (Glanz and Bishop 2010). The Capability, Opportunity, Motivation and Behaviour (COM‐B) model is a theoretical framework developed from theories of behaviour change. It forms the central component of the Behaviour Change Wheel (Michie et al. 2014), a theory‐driven process for developing and evaluating complex interventions. The central principle of the model is that for behaviour to take place, there must be capability (physical or psychological, to engage in a behaviour), opportunity (physical and social, to support a behaviour) and motivation (reflective or automatic, to complete the behaviour). The Theoretical Domains Framework (TDF) (Cane et al. 2012) builds on the components of the COM‐B and can be used to further define the barriers and enablers to RCB (see Figure 1). The TDF is an integrated theoretical framework which includes 14 domains, covering 84 theoretical constructs (Atkins et al. 2017). The framework has been applied across healthcare contexts such as hand hygiene compliance (Dyson et al. 2011) and offers a robust framework for exploring RCC.

Publications that explore RCC in speech and language therapy specifically are limited. Finch et al. (2013) evaluated the research interest, experience and confidence of SLTs working in Australia. Results indicated increased confidence with basic research activities, for example, those needed for evidence‐based practice (EBP) and lower confidence with more advanced research activities, for example, analysing and publishing results (Finch et al. 2013). Other studies have included SLTs as part of a wider AHP group, largely conducted within the Australian healthcare system (e.g., Alison et al. 2017; Lee et al. 2020). In the UK National Health Service (NHS), Dickens et al. (2024) evaluated RCC in a mental health and disability trust, which included a small number of SLTs (*n* = 20, 6.8% of total staff group). They found that RCC was lower in all domains but two when compared to prior evaluations in physical health contexts. Cordrey et al. (2022) and Luckson et al. (2018) found that barriers and enablers differed by level, for example, individual, team or organisational levels, but also noted a lack of understanding around individual level barriers. Comer et al. (2022) conducted a national cross‐sectional survey, which included 328 SLTs (9.8% of the total possible participants) and identified time constraints, limited research training and unclear clinical‐academic career pathways as key barriers. However, SLTAs were not included in any of the above studies, and none applied the COM‐B or TDF.

While the SLT profession has made notable progress in developing research capacity and leadership, often in advance of some other AHPs, there remain important gaps, particularly in underrepresented specialisms such as mental health and disability, where research infrastructure and leadership are less well established (Dickens et al. 2024). In these contexts, there is a clear need to focus on individual‐level engagement in research to identify the specific barriers and enablers encountered by speech and language therapy staff. To the authors’ knowledge, no previous study has applied the COM‐B model and TDF specifically to SLTs and SLTAs within an NHS Trust to explore these factors. This study aims to address this gap by investigating the individual‐level influences on research activity within a speech and language staff group in an NHS setting. The findings aim to inform the development of a tailored RCB strategy.

The aims of this study are to

1. Identify the barriers and enablers to research activity for speech and language therapy staff within a large mental health and disability NHS Trust (NHS Trust), utilising behaviour change theory as a framework for study design and analysis.
2. Establish what speech and language therapy staff within an NHS Trust deem to be their current level of research engagement, utilising the categories developed within the IPF (Whitworth et al. 2012).

This study is reported in line with the Checklist for Reporting of Survey Studies (CROSS) (Sharma et al. 2021), see Supporting File 1.

## 2 Methods

The study design was based upon the six stages outlined by Atkins et al. (2017).

### 2.1 Step 1: Select and Specify the Target Behaviours

Related to research engagement and RCC, the behaviour identified was research‐related activities linked to the individual, team and organisation. This included a focus on early research development skills, such as those required to achieve standards 11.1, 11.2, 11.3 of the HCPC standards (Health Care Professions Council 2024). In this study, ‘speech and language therapy staff’ were defined as the SLTs and SLTAs employed within the speech and language therapy service, as a coherent group.

### 2.2 Step 2: Select the Study Design

A cross‐sectional survey was conducted.

### 2.3 Step 3: Decide the Sampling Strategy

Eligibility criteria:

1. Participants were either qualified SLTs registered with the HCPC or SLTAs.
2. Participants were employed by the speech and language therapy department of the NHS Trust where the study was conducted.

A purposive sampling procedure was used to identify all who fulfilled the inclusion criteria. Permission to attend staff meetings to advertise the study was sought from the professional lead and the manager of speech and language therapy. Recruitment and data collection took place between November and January 2024. A multipronged approach was utilised with the first author attending meetings remotely through an online platform (Microsoft Teams) to share information. A video recording of the first author advertising the study was sent to staff via communication emails to increase engagement, with email reminders sent periodically.

This research‐active NHS Trust provided inpatient and community services and had an established relationship with a Higher Education Institution (HEI) offering speech and language therapy degree training and student placements. Speech and language therapy staff received training to support service evaluations, were invited to attend an annual research conference and had access to funded research internships from the NHS Trust. This research infrastructure formed part of the context for participant recruitment and may be relevant when considering the study setting.

### 2.4 Step 4: Develop the Data Collection Tool

The COM‐B model (Michie et al. 2014) and TDF were used as the basis to create the survey. Questions were developed, focussed on each TDF domain, guided by literature on RCC and informed by the RCCT (Holden et al. 2012). The domain of physical capability was omitted as not relevant in this context. Questions were structured to be answered using fixed alternatives of ‘Yes’, ‘No’ or ‘Don't Know’. One free‐text question was included for the COM‐B component of ‘Automatic Motivation’ (see Supporting File 2). Finally, participants were invited to rate their level of research engagement using categories from the IPF (Whitworth et al. 2012).

Demographic data included clinical specialism (grouped to protect identity), pay band, years since qualification, highest qualification, research history, inclusion of research in job role, time allocated to research and whether research activity is discussed in appraisals. No personally identifiable information was collected. The survey was piloted for accessibility and language with SLTs external to the trust. All questionnaire items were mandatory, except for Questions 6, 8 and 15. These items were conditional follow‐up questions, presented only to participants who provided a positive response to the preceding item and were designed to elicit additional information where relevant.

### 2.5 Step 5: Collect the Data

A URL to the online survey (via Microsoft forms) was distributed to speech and language therapy staff by email following an advertisement. Participants were provided with study information, including aims, ethical approval, data management and contact details. Participation was voluntary, anonymous and required confirmation of consent and eligibility before accessing the 15‐min survey. Participants were given 28 days to complete the survey. Up to three reminder emails were sent at 14‐day intervals following this initial period.

### 2.6 Step 6: Analyse the Data

Quantitative data were analysed using descriptive statistics. Percentage of ‘Yes’, ‘No’ and ‘Don't Know’ responses was used to classify each RCC component as an enabler, barrier or unknown. For example, ‘Yes’ to ‘Allocated time to be involved in research?’ indicated an enabler; a ‘No’ a barrier and ‘Don't know’ uncertainty. Total scores were categorised as weak (0%–33.33%), moderate (33.34%–66.66%) and strong (66.67%–100%). Deductive content analysis was applied to the free text responses (Elo and Kyngäs 2008), organised using NVivo (v12.0) and guided by a structured matrix of barriers or enablers. Emotionally positive words were coded as enablers, negative as barriers. Responses were categorised, percentages calculated and coding was conducted by Author 1 and independently checked by Author 2 through an iterative consensus process.

### 2.7 Ethical Approval

Ethical approval was gained from the NHS Trust (SER‐23‐208). The study was registered with the NHS Trust through which it was being conducted who classified it as a Service Evaluation. Health Research Authority and NHS ethical approval were not required as the study recruited only staff.

## 3 Results

### 3.1 Participant Demographic Data

Fifty‐seven (response rate 73.08%) of a total 78 speech and language therapy staff members within the organisation completed the survey. These comprised 52 of 66 SLTs and five of 12 SLTAs. All participants who started the survey finished it. See Table 1 for details.

**TABLE 1:** Demographic data of participants.

Question	Number	Percentage (%)
Area of clinical specialism	(*n* = 71)	
(Staff could select up to two areas if working across specialisms)		
SLTAs		
Adult learning disability	2	2.82
Dysphagia	1	1.40
Paediatric learning disability	3	4.23
Paediatric mental health	1	1.40
SLTs		
Adult learning disability	18	25.35
Adult neurorehabilitation/neuropsychiatry	8	11.27
Adult psychiatry services	10	14.09
Augmentative and alternative communication	4	5.63
Dysphagia	0	0.00
Neurodiversity	7	9.86
Paediatric learning disability	9	12.68
Paediatric mental health	6	8.45
Voice	2	2.82
Pay band	(*n* = 57)	
SLTAs		
Bands 2–4	5	8.77
SLTs		
Band 5 or 6	23	40.35
Bands 7, 8a, 8b, 8c	29	50.88
Number of years since qualifying as a SLT	(*n* = 57)	
SLTAs		
0–5 years	2	3.51
No answer	3	5.26
SLTs		
0–5 years	20	35.09
6–10 years	14	24.56
11–15 years	7	12.28
16–20 years	7	12.28
20 years+	4	7.02
Highest level of qualification	(*n* = 57)	
SLTAs certificate/diploma		
Degree	2	3.51
SLTs	3	5.26
Degree	23	40.35
Masters/Postgraduate	29	50.88
Doctoral	0	0.00
Did you conduct a research study as part of previous training?	(*n* = 57)	
SLTAs		
Yes	2	3.51
No	1	1.75
Don't know	0	0.00
Not applicable	2	3.51
SLTs		
Yes	40	70.18
No	9	3.51
Don't know	2	1.75
Not applicable	1	
Are you enrolled in any research‐related training?	(*n* = 57)	
SLTAs		
Yes	1	1.75
No	4	7.02
SLTs		
Yes	6	10.53
No	46	80.70
If yes, what level of study are you enrolled In?	(*n* = 7)	
SLTAs		
In‐work training	1	1.75
SLTs		
In‐work training	3	5.26
Postgraduate certificate/diploma	3	5.26
Is research engagement/activity discussed within your appraisal?	(*n* = 57)	
SLTAs		
Yes, routinely	2	3.51
Only if I am involved in research or asked to discuss it	1	1.75
No	2	3.51
SLTs		
Yes, routinely	9	15.79
Only if I am involved in research or asked to discuss it	33	57.89
No	10	17.54
Are research‐related activities part of your job role?	(*n* = 57)	
SLTAs		
Yes	2	3.51
No	1	1.75
Don't know	2	3.51
SLTs		
Yes	22	38.60
No	16	28.07
Don't know	14	24.56
Amount of time in current role that is formally allocated to research or research‐related activity	(*n* = 57)	
SLTAs		
Less than 25% of time	4	7.02
More than 25% but less than 50%	1	1.75
More than 50% but less than 75%	0	0.00
More than 75% of my time	0	0.00
SLTs		
Less than 25% of time	50	87.7
More than 25% but less than 50%	2	23.51
More than 50% but less than 75%	0	0.00
More than 75% of my time	0	0.00

### 3.2 Barriers and Enablers to Research Activity

**FIGURE 2:**
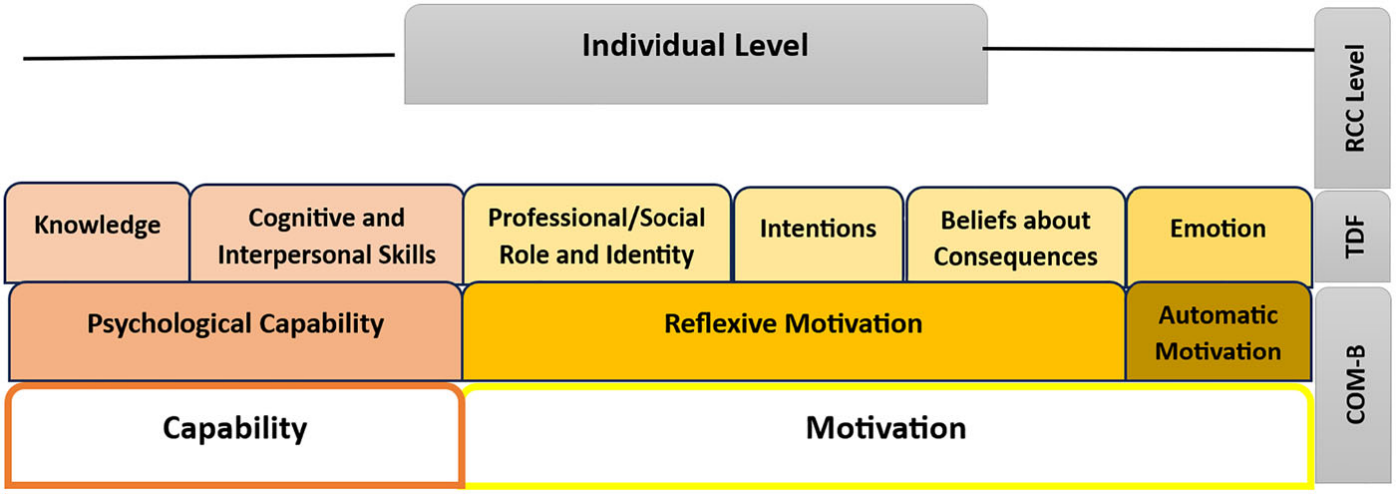
COM‐B components and TDF domains represented at the individual level of RCC.

Participants rated their perceived capabilities, opportunities and motivations for research activity across eight of the 14 TDF domains, which were categorised as barriers or enablers (Figures 2 and 6). The domains, such as knowledge, cognitive and interpersonal skills, environmental context and resources, social influences, professional/social sole and identity, intentions, beliefs about consequences and emotion, are reported in relation to individual, team and organisational levels. A full summary of item level responses for each domain is provided in Supporting File 3.

### 3.3 Individual Level Barriers and Enablers

#### 3.3.1 Capability: Psychological (TDF Domains: Knowledge; Cognitive and Interpersonal Skills)

Psychological capability was assessed through perceived barriers and enablers in tasks such as EBP, monitoring and evaluation, service evaluation and audit and advanced research activities. Strengths were classified using percentage thresholds outlined in the methods. Findings are reported for both knowledge and cognitive/interpersonal skills domains at the individual level.

##### 3.3.1.1 Evidence‐Based Practice Activities

Searching the literature was reported as a strong enabler for knowledge (91.23%) and for skills (85.96%). Critically appraising literature was also a strong enabler (77.19% for knowledge; 73.68% for skills). Using referencing software was rated as a moderate enabler (61.40% for knowledge; 59.65% for skills).

##### 3.3.1.2 Monitoring and Systematically Evaluating Practice

Designing a way to collect qualitative data was evaluated as a strong enabler for both knowledge (77.19%) and skills (66.67%). Designing a way to collect quantitative data was a strong enabler for knowledge (66.67%) and a moderate enabler for skills (56.14%).

##### 3.3.1.3 Service Evaluation and Audit

Designing a service evaluation was identified as a strong enabler in knowledge (68.43%) and a moderate enabler in skills (57.89%). Designing an audit was rated as a moderate enabler in both knowledge (53.39%) and skills (49.12%). Registering a service evaluation or audit was reported as a moderate barrier in knowledge (57.89%) and skills (50.88%).

##### 3.3.1.4 More Advanced Research Activities

**FIGURE 3:**
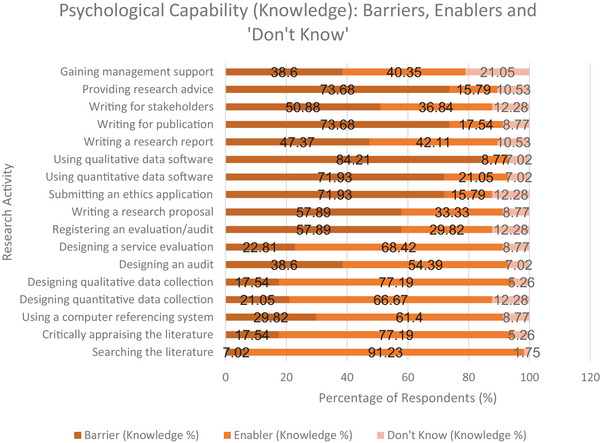
Psychological Capability (Knowledge) as Perceived Barriers, Enablers, and ‘Don't Know’ Responses Across Research Activities.

**FIGURE 4:**
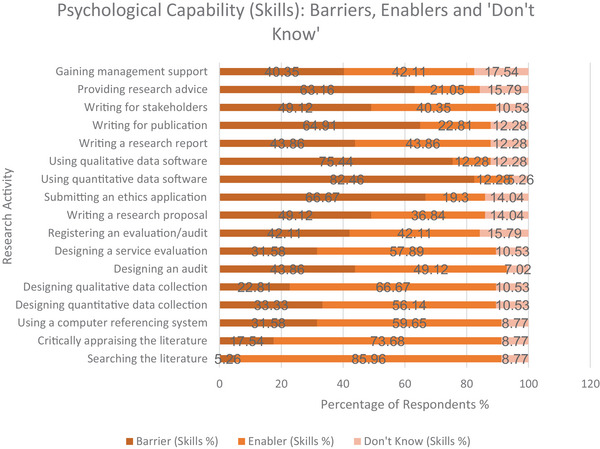
Psychological capability (skills) as perceived barriers, enablers and ‘Don't Know’ responses across research activities.

Submitting an ethics application was rated as a strong barrier for knowledge (71.93%) and skills (66.67%). Using quantitative data analysis software was rated as a strong barrier in knowledge (71.93%) and skills (82.46%). Using qualitative data analysis software was also rated as a strong barrier (84.21% for knowledge; 75.44% skills). Writing for publication in a peer‐reviewed journal was reported as a strong barrier in knowledge (73.68%) and skills (64.91%). Providing advice to others about research‐related activities was identified as a strong barrier in knowledge (73.68%) and skills (63.16%). Figures 3 and 4 provide a visual summary of findings.

#### 3.3.2 Motivation: Reflexive and Automatic

##### 3.3.2.1 Reflexive (TDF Domain: Professional/Social Role and Identity; Intentions; Beliefs About Consequences)

**FIGURE 5:**
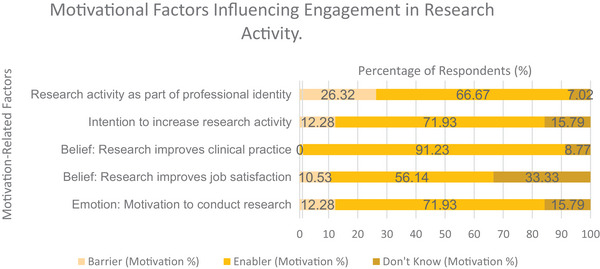
Reflexive and automatic motivation as perceived barriers, enablers and ‘Don't Know’ responses for engagement in research activities.

Reflexive motivation was assessed across three TDF domains. All items were more frequently rated as enablers than barriers (Figure 5).

Intention to increase research participation was a strong enabler (71.93%), as was the belief that research improves clinical practice (91.23%). Two‐thirds (66.67%) viewed research activity as part of their job role and professional identity, both of which were strong enablers. Belief that research enhances job satisfaction was a moderate enabler by 56.14%, though 33.33% responded ‘don't know’.

##### 3.3.2.2 Automatic (TDF Domain: Emotion)

Automatic motivation was explored through the TDF domain of Emotion. A strong enabler was reported by 71.93% of participants, who felt motivated by conducting research into their clinical specialism. In free‐text responses, 42.11% reported both enabler and barrier emotions, 26.32% only enablers and 31.58% only barriers (see Supporting File 4). The most frequently reported enabler emotions were ‘excited’ (22.81%), ‘motivated’ (15.79%) and ‘interested’ (12.28%); common barrier emotions included ‘overwhelmed’ (21.05%), ‘nervous’ (10.53%) and ‘daunted’ (8.77%).

### 3.4 Team and Organisational Level Barriers and Enablers

#### 3.4.1 Opportunity: Physical (TDF Domains: Environmental Context and Resources)

**FIGURE 6:**
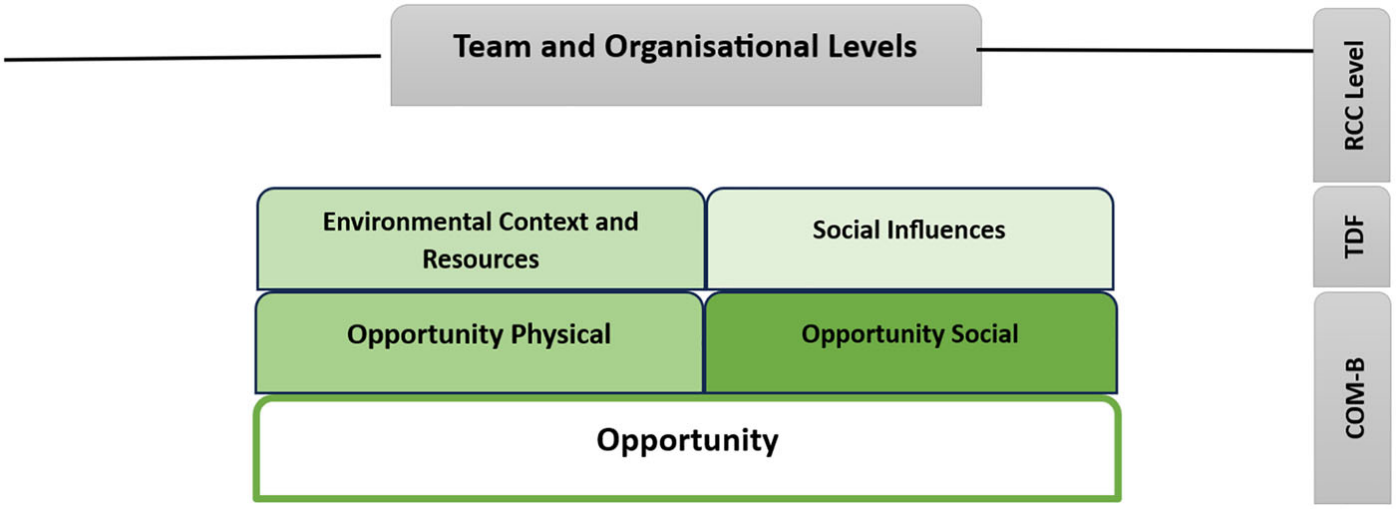
COM‐B components and TDF domains represented at the team and organisational levels of RCC.

**FIGURE 7:**
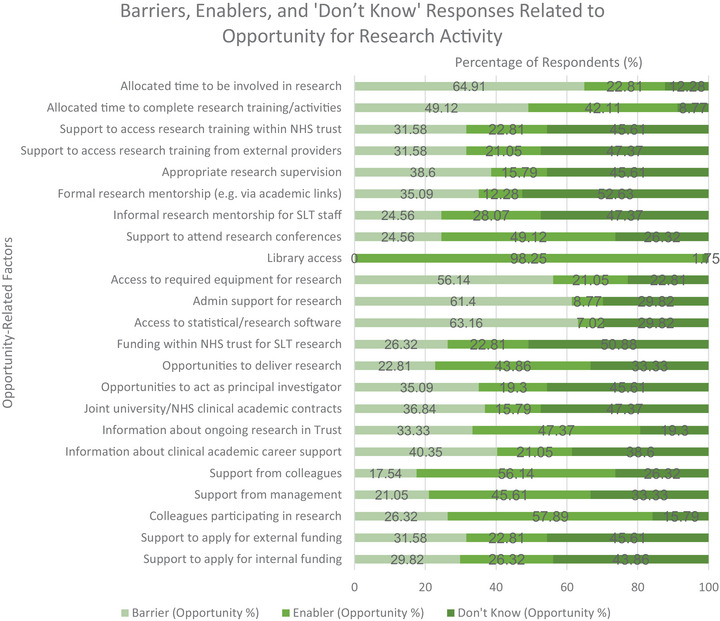
Percentage of respondents reporting barriers, enablers and ‘Don't Know’ responses related to physical and social opportunity for research engagement.

Physical opportunity was assessed through items on time, opportunities for skill development and mentoring, resources and infrastructure, funding and job opportunities. As responsibility spanned team and organisational levels, responses could not be separated (see Figure 6). A stacked bar chart illustrates percentages of barriers, enablers and ‘don't know’ responses across this domain (Figure 7).

##### 3.4.1.1 Time

Lack of time for research was a moderate barrier for 64.91%. Support to attend research conferences was a moderate enabler (49.12%). Allocated time for research training/activities was rated both a moderate enabler (42.11%) and a barrier (49.12%). Supervision and mentorship were moderate barriers (38.60% and 35.09%, respectively). Several items in this domain had the highest proportion of ‘don't know’ responses: access to internal training (45.61%), external training (47.37%) and informal research supervision or mentorship (47.37%).

##### 3.4.1.2 Resources and Infrastructure

Library access was a strong enabler (98.25%). Other infrastructure‐related items were moderate barriers: equipment (56.14%), admin support (61.40%) and access to software or statistical packages (63.16%).

##### 3.4.1.3 Funding and Job Opportunities

Opportunities to deliver research were rated as a moderate enabler (43.86%). All other items received the highest percentage of ‘don't know’ responses: internal funding (50.88%), principal investigator roles (45.61%) and joint university/NHS Trust contracts (47.37%).

#### 3.4.2 Opportunity: Social (TDF Domain: Social Influences)

Social opportunity was assessed through access to research information and social support.

##### 3.4.2.1 Research Information

Information about ongoing research projects in the NHS Trust was a moderate enabler for 47.37% of participants. In contrast, information about how the NHS Trust supports or promotes CA careers was a moderate barrier for 40.35%.

##### 3.4.2.2 Social Support

Moderate enabler included support from colleagues (56.14%), management (45.61%) and having research‐active colleagues (57.89%). The remaining factors had the highest proportion of participants stating, ‘don't know’. These were encouragement/support to apply for external (45.61%) and internal (43.86%) research funding.

##### 3.4.2.3 Level of Research Engagement

**FIGURE 8:**
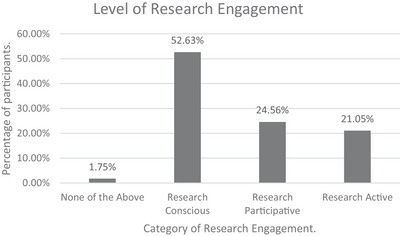
Self‐reported level of research engagement.

The majority of participants identified as ‘Research Conscious’ (52.63%), followed by ‘Research Participative’ (24.56%) and ‘Research Active’ (21.05%). One participant selected none of the listed categories (see Figure 8).

## 4 Discussion

No studies have previously examined barriers and enablers to RCB for NHS speech and language therapy staff using the Capability, Opportunity, Motivation‐Behaviour (COM‐B) model and TDF. This study addressed this gap by analysing RCC at an individual level focussing on capabilities aligned to the HCPC SoP for SLTs (Health Care Professions Council 2024; SoP 11.1, 11.2 and 11.3). Barriers and enablers were coded into eight TDF domains corresponding to COM‐B components. Individual level domains included knowledge, cognitive and interpersonal skills, professional/social role and identity, intentions, beliefs about consequences and emotion. Team and organisational level domains were environmental context and resources, and social influences. Participants also completed a self‐assessment of research engagement using the IPF (Whitworth et al. 2012).

Utilising the COM‐B and TDF enabled identification of priority areas for intervention. Speech and language therapy staff showed strong foundational knowledge and skills supporting research engagement. However, advanced research tasks were frequently rated as barriers, highlighting the need for both organisational support and individual motivation.

Knowledge and skills to complete EBP, such as searching and critically appraising the literature, were strong enablers. This is consistent with findings of previous studies where knowledge and skills developed at an early stage of research training were more established for AHPs (Holden et al. 2012; Alison et al. 2017). This is perhaps less surprising as such skills are generally acquired during training as requirements for professional registration and are integral to the job role (Health Care Professions Council 2024). Participants also felt confident gathering and using qualitative and quantitative feedback. National directives have placed an expectation on health professionals to seek and include the patient's perspective during care and to use this to evaluate the care offered (NHS England 2019). It may be concluded that the clinical context has supported the development of the knowledge and skills required to a level where staff felt confident to use them in practice.

When considering the evaluation of practice, one activity (knowledge to design a service evaluation) was a strong enabler for knowledge and a moderate enabler for skills. Within the context of the study, the local HEI (Newcastle University) offers student time, education and mentoring to SLTs to conduct a service evaluation within clinical teams. For AHPs, where less priority is placed upon time and resource for research compared to clinical work (Comer et al. 2022; Cordrey et al. 2022), the offer of additional resources through links with a student SLT is likely to be highly valued. Audit design, however, was rated more variably, suggesting disparity in knowledge between participants. It is an organisational requirement to register audit activities, yet few stated they had the knowledge to do this. The expectations for SLTs to participate in regular clinical audit are reinforced by publications (Burgess and Moorhead 2011) alongside legally required SoPs set out by the regulatory body (Health Care Professions Council 2024). These findings point to a need for targeted training.

For more advanced research activities, participants identified more barriers than enablers, contrasting with the stronger enablers reported for early‐stage EBP tasks. This aligns with existing literature highlighting that AHPs often lack the research skills, experience and knowledge required for later‐stage research engagement (e.g., Cordrey et al. 2022; Finch et al. 2013; Alison et al. 2017). Activities such as writing research reports, disseminating findings to stakeholders and gaining managerial support were rated as moderate for both barriers and enablers. Other advanced research tasks had more participants marking them as barriers, further suggesting gaps in capability. Many participants had completed research as part of undergraduate training, so it was expected that this would be an enabler. Comer et al. (2022) found that clinicians can become ‘rusty’ (p. 4) through a lack of regular involvement in research. Level of qualification for participants should also be considered due to the link between this and research skill (Alison et al. 2017; Finch et al. 2013). In this study, the highest qualification level was master's/postgraduate, with no participants having achieved a doctoral level or being enrolled in master's, doctoral level or postdoctoral level research training.

Motivational factors, reflected in the TDF domains of professional/social role and identity, intentions, beliefs about consequences and emotion, played a key role. AHPs have previously shown a high level of interest in undertaking research (Lazzarini et al. 2013). In this study, like others (Comer et al. 2022; Pager et al. 2012), participants expressed a strong desire to increase their research activity and believed it improves clinical practice. Such a consistently held belief amongst a staff group links to the ‘support for research activity from colleagues’ expressed within the social influences’ domain. Pager et al. (2012) found that a strong interest, job satisfaction, opportunity to develop skills and to improve practice are all factors which underlie increased motivation to engage in research for AHPs. They suggested that providing opportunities to engage in research was an important source of job satisfaction and a driver for improvement and change. Not all participants felt that job satisfaction would improve if they engaged in research; it is interesting to consider what may have influenced this belief. Cordrey et al. (2022) found that a lack of motivation may be linked to a lack of time to engage in research activity, seeing research as a barrier to maintaining a work–life balance. This was supported by Pager et al. (2012) and Alison et al. (2017).

The belief that research activity is linked to job role and professional identity was only moderately held. To foster a positive research culture, research needs to be embedded at team, organisational and network levels to reinforce its value (Golenko et al. 2012; Wenke et al. 2020). At the network level, the RCSLT and HEE list RCC as a priority (Royal College of Speech and Language Therapy 2022a, 2022b; Health Education England 2022), and many organisations list research as a strategic aim. However, no strategy exists at the team level for this group of participants. Emotional responses were mixed; a moderate number expressed both negative and positive emotions. Comer et al. (2022) found that some participants had feelings of despondency, driven by uncertainty about how to undertake research, which may be a factor here.

At the team and organisational levels, a lack of time to engage in research activities was a moderate barrier. This is consistent with wider AHP literature, with many expressing conflicts between clinical demands and research productivity (Comer et al. 2022; Pager et al. 2012; Luckson et al. 2018). Consequently, conducting research in the clinical setting can be perceived as too difficult to achieve. Other contributing factors are as follows: competing demands, workload pressures, staffing issues and lack of backfill (Pager et al. 2012; Luckson et al. 2018). In a study conducted by Cordrey et al. (2022), 29 participants had research as part of their role description, yet only six had dedicated time to do it, suggesting that including research within job descriptions alone is not enough to enable research to happen.

Support for SLT staff to attend research conferences provides an opportunity for development and mentoring and was a moderate enabler. This is likely to be influenced by access to a regional research network that supports SLT staff, providing a showcase for local research and an opportunity for networking and support for research development. This emphasises the importance of understanding the specific context in which the phenomenon is occurring. However, access to support, research training, mentorship and funding was inconsistent. Many participants selected ‘don't know’, suggesting limited awareness of existing opportunities. Luckson et al. (2018) highlighted that poor communication can act as a barrier to RCB when organisational‐level messages do not reach individual staff. This study supports that finding, with some participants unaware of available support. Participants felt that there was a moderate barrier for research supervision, including through academic links, again with a high percentage selecting ‘don't know’. A lack of access to dedicated mentorship can act as a significant barrier to RCB (Cordrey et al. 2022). This corresponds with people reporting weak enablers and a significant lack of knowledge for encouragement and/support to apply for research funding (internal and external). Development of mentorship opportunities, such as the research champions’ network created by the RCSLT, is essential to building research infrastructure and research career development (Westwood et al. 2018). Collaborations with HEI can progress research infrastructure, such as support to apply for research funding and CA career pathways (Westwood et al. 2018).

Within the TDF social influences domain, support from management was rated as a moderate enabler, with a third of participants stating they ‘don't know’ if management supported research. It could be that support for research from middle management varies as it is delivered at an individual level. Studies have suggested that strategies at the organisational level may not adequately connect with the individual level (Comer et al. 2022) due to dependency on middle managers to implement them in the context of daily practice. Middle managers may themselves lack research skills or a clear understanding of the priority placed upon research by senior managers when compared to other priorities, such as managing clinical demands.

For resources and infrastructure, library access was the main enabler, present for all participants except one. This contrasted with other resources, such as equipment, admin support and access to statistical software, all of which were rated as a moderate barrier. Failure to access supportive infrastructure is a common barrier nationally in the United Kingdom for AHPs (Comer et al. 2022; Alison et al. 2017; Pager et al. 2012). These results suggest a lack of dedicated funding and resources at the team and organisation levels. The highest percentage of participants had no knowledge of the funding and job opportunities available to support staff as active researchers. Again, suggesting a breakdown in communication and a lack of visible, team‐level research job opportunities. This was further illustrated with participants rating the provision of information about how the NHS Trust supports/promotes CA careers as a moderate barrier. A lack of understanding regarding CA roles, coinciding with the absence of established career pathways are barriers for AHPs nationally (Comer et al. 2022). Research growth and leadership can be limited by the scarcity of AHP CA roles. At the organisational and team level, the establishment of CA roles has not been commonly adopted (Newington et al. 2021). These results suggest the need for the development of CA roles within speech and language therapy to build opportunities for research and mentorship and to support the development of research leadership and education (Cane et al. 2012).

In this study, all but one participant reported some level of research engagement, as classified by the IPF (Whitworth et al. 2012). The distribution closely reflected the original IPF model; the largest group identified as ‘Research Conscious’, indicating they had the knowledge and skills to meet HCPC SoP; fewer were ‘Research Participative’, and the smallest ‘Research Active’. This is as expected in a service where clinical work is the central activity, informed by knowledge of the evidence base (research conscious). There may be an opportunity for practitioners to take part in research projects originating from within or outside the team/organisation (research participative), and a small number of highly motivated staff may have funding to conduct primary research whilst their clinical role is backfilled. Furthermore, these roles are fluid, shifting and changing circumstances, interests and opportunities. While not investigated in this study, future research could explore whether factors such as years of experience or banding influence research engagement. For instance, newly qualified SLT staff may be more research aware due to recent training. Such insights could inform targeted, clinically relevant training and RCB initiatives.

### 4.1 Strengths and Limitations

The results of this survey should be considered in the context of the following limitations and strengths. First, the response rate from participants as a percentage of the staff group invited to take part is large and greater than that previously published in studies investigating RCC (Cordrey et al. 2022; Luckson et al. 2018; Finch et al. 2013). It should be noted, however, that, as with all surveys, the respondents may be affected by selection bias, with greater representation from those who consider research to be linked to their role. Furthermore, a cross‐sectional survey is designed to provide data at a particular point in time and therefore cannot show changes over time. Despite this, the response rate is high and should be taken as a fair representation of the views of speech and language therapy staff in the NHS Trust surveyed.

The COM‐B and TDF provide a psychologically informed understanding of barriers and enablers to research activity for this context and the areas of RCC included. The questionnaire placed focus on the individual level, with some team and organisational factors. This enabled a deeper evaluation at this level, important to support the development of an RCB strategy at the individual and team levels. However, a limitation of this focus on individual‐level factors is the relative lack of in‐depth exploration of organisational‐level influences. The absence of detailed consideration of broader structural and cultural factors within the organisation limits understanding of how these may also enable or restrict research activity. Furthermore, RCB frameworks identify other influential markers, such as the number of publications or the value of funding awarded for research. Inclusion of factors such as these alongside the data collected here may be helpful for the measurement of RCB success.

## 5 Conclusions

Speech and language therapy staff who responded to this survey was highly motivated to engage in research, with a desire to increase the amount of research in their clinical practice. This was supported by positive beliefs about the beneficial influence research has on patient care and reports of colleague support. Their knowledge and skills to complete activities related to EBP were a strong enabler to research engagement. Knowledge and skills to complete other activities required to comply with HCPC SoP varied across the group. Knowledge and skills to complete more advanced research activities were an overall barrier. A lack of skills and knowledge for more advanced research activities has consistently been reported by AHPs as a barrier to RCB (Cordrey et al. 2022; Finch et al. 2013; Pager et al. 2012; Alison et al. 2017). However, clear training priorities can be identified in this study. Participants reported barriers in the environmental context and resources domain as a lack of time, appropriate supervision, admin support and access to software to support research. Many said they lacked understanding of whether supervision, support, funding or CA job opportunities were available to them, signalling a breakdown in communication at the team level and a lack of visible team‐level role models, mentors and research support networks. The influence of a network‐level support from a HEI was evident in participants’ knowledge and skills to complete service evaluation and the opportunity to attend research‐focused conferences. The information gained from this study has some relevance to other speech and language therapy services by highlighting areas where barriers and enablers may exist in this professional group.

Based on these findings, clinical managers should consider embedding research expectations and pathways more clearly within SLT team roles, improving communication about available resources and prioritising dedicated research time and supervision structures. Educators and HEIs can contribute by strengthening partnerships with SLT teams to provide mentoring, student‐supported evaluations and clinically relevant research training. Developing clear local strategies to align organisational research goals with individual and team‐level support is essential. Future research could examine how factors such as staff banding, professional experience or access to CA pathways influence research engagement. Longitudinal studies could also explore the impact of targeted RCB interventions over time.

## Supporting information




**Supplementary File 1**: Checklist for Reporting of Survey Studies (CROSS)

**Supplementary File 2**: Research Capacity and Culture in Speech and Language Therapy Staff Survey.

**Supplementary File 3**: Barriers and Enablers Data from Participant Questionnaire.

**Supplementary File 4**: Respondents’ Emotions When Thinking About Research

## Data Availability

The data that support the findings of this study are not publicly available due to the confidentiality agreement with participants, as outlined in the consent process. Data are stored securely for 5 years in accordance with ethical approval requirements and are accessible only to the research team.

## References

[jlcd70116-bib-0001] Alison, J. A. , B. Zafiropoulos , and R. Heard . 2017. “Key Factors Influencing Allied Health Research Capacity in a Large Australian Metropolitan Health District.” Journal of Multidisciplinary Healthcare 10: 277–291. 28860795 10.2147/JMDH.S142009 PMC5558427

[jlcd70116-bib-0002] Atkins, L. , J. Francis , R. Islam , et al. 2017. “A Guide to Using the Theoretical Domains Framework of Behaviour Change to Investigate Implementation Problems.” Implementation Science 12: 1–18. 28637486 10.1186/s13012-017-0605-9 PMC5480145

[jlcd70116-bib-0003] Burgess, R. , and J. Moorhead . 2011. New Principles of Best Practice in Clinical Audit. Radcliffe Publishing.

[jlcd70116-bib-0004] Burr, S. 2020. “Understanding Allied Health Professional (AHP) Clinical Academic (CA) Roles.” In Solent NHS Trust Research & Improvement Conference . 10.13140/RG.2.2.15216.89608/1.

[jlcd70116-bib-0005] Cane, J. , D. O'connor , and S. Michie . 2012. “Validation of the Theoretical Domains Framework for Use in Behaviour Change and Implementation Research.” Implementation Science 7: 1–17. 10.1186/1748-5908-7-37 PMC3483008 22530986

[jlcd70116-bib-0006] Comer, C. , R. Collings , A. Mccracken , C. Payne , and A. Moore . 2022. “Allied Health Professionals' Perceptions of Research in the United Kingdom National Health Service: A Survey of Research Capacity and Culture.” BMC Health Services Research 22: 1094. 36030236 10.1186/s12913-022-08465-6 PMC9420271

[jlcd70116-bib-0007] Condell, S. L. , and C. Begley . 2007. “Capacity Building: A Concept Analysis of the Term Applied to Research.” International Journal of Nursing Practice 13: 268–275. 17883712 10.1111/j.1440-172X.2007.00637.x

[jlcd70116-bib-0008] Cooke, J. 2005. “A Framework to Evaluate Research Capacity Building in Health Care.” BMC Family Practice 6: 44. 16253133 10.1186/1471-2296-6-44 PMC1289281

[jlcd70116-bib-0009] Cooke, J. , P. Gardois , and A. Booth . 2018. “Uncovering the Mechanisms of Research Capacity Development in Health and Social Care: A Realist Synthesis.” Health Research Policy and Systems 16: 1–22. 30241484 10.1186/s12961-018-0363-4 PMC6150992

[jlcd70116-bib-0010] Cordrey, T. , E. King , E. Pilkington , K. Gore , and O. Gustafson . 2022. “Exploring Research Capacity and Culture of Allied Health Professionals: A Mixed Methods Evaluation.” BMC Health Services Research 22: 85. 35039018 10.1186/s12913-022-07480-x PMC8764821

[jlcd70116-bib-0011] Department of International Development . 2010. “Capacity Building in Research. How to Note.” A DFID Practice Paper. Microsoft Word—HTN Capacity Building Final 21 06 10.doc. publishing.service.gov.uk.

[jlcd70116-bib-0012] Dickens, G. L. , M. Avantaggiato‐Quinn , S.‐J. Long , M. Schoultz , and N. Clibbens . 2024. “Mental Health Nurses' and Allied Health Professionals' Individual Research Capacity and Organizational Research Culture: A Comparative Study.” SAGE Open Nursing 10: 23779608241250207. 38746076 10.1177/23779608241250207 PMC11092560

[jlcd70116-bib-0013] Dyson, J. , R. Lawton , C. Jackson , and F. Cheater . 2011. “Does the Use of a Theoretical Approach Tell Us More About Hand Hygiene Behaviour? The Barriers and Levers to Hand Hygiene.” Journal of Infection Prevention 12: 17–24.

[jlcd70116-bib-0014] Elo, S. , and H. Kyngäs . 2008. “The Qualitative Content Analysis Process.” Journal of Advanced Nursing 62: 107–115. 18352969 10.1111/j.1365-2648.2007.04569.x

[jlcd70116-bib-0015] Finch, E. , P. Cornwell , E. C. Ward , and S. M. Mcphail . 2013. “Factors Influencing Research Engagement: Research Interest, Confidence and Experience in an Australian Speech‐Language Pathology Workforce.” BMC Health Services Research 13: 1–11. 23597184 10.1186/1472-6963-13-144 PMC3637558

[jlcd70116-bib-0016] Glanz, K. , and D. B. Bishop . 2010. “The Role of Behavioral Science Theory in Development and Implementation of Public Health Interventions.” Annual Review of Public Health 31: 399–418. 10.1146/annurev.publhealth.012809.103604 20070207

[jlcd70116-bib-0017] Golenko, X. , S. Pager , and L. Holden . 2012. “A Thematic Analysis of the Role of the Organisation in Building Allied Health Research Capacity: A Senior Managers' Perspective.” BMC Health Services Research 12: 1–10. 22920443 10.1186/1472-6963-12-276 PMC3464180

[jlcd70116-bib-0018] Health Care Professions Council . 2024. “The Standards of Proficiency for Speech and Language Therapists.” Speech and Language Therapists | (hcpc‐uk.org).

[jlcd70116-bib-0019] Health Education England . 2022. “Allied Health Professions' Research and Innovation Strategy for England.” HEE Allied Health Professions Research and Innovation Strategy.

[jlcd70116-bib-0020] Health Research Authority . 2021. “UK Policy Framework for Health and Social Care Research.” Health Research Authority. https://www.hra.nhs.uk.

[jlcd70116-bib-0021] Holden, L. , S. Pager , X. Golenko , and R. S. Ware . 2012. “Validation of the Research Capacity and Culture (RCC) Tool: Measuring RCC at Individual, Team and Organisation Levels.” Australian Journal of Primary Health 18: 62–67. 22394664 10.1071/PY10081

[jlcd70116-bib-0023] Jonker, L. , and S. J. Fisher . 2018. “The Correlation Between National Health Service Trusts' Clinical Trial Activity and Both Mortality Rates and Care Quality Commission Ratings: A Retrospective Cross‐Sectional Study.” Public Health 157: 1–6. 29438805 10.1016/j.puhe.2017.12.022

[jlcd70116-bib-0024] Jonker, L. , S. J. Fisher , and D. Dagnan . 2020. “Patients Admitted to More Research‐Active Hospitals Have More Confidence in Staff and Are Better Informed About Their Condition and Medication: Results From a Retrospective Cross‐Sectional Study.” Journal of Evaluation in Clinical Practice 26: 203–208. 30784152 10.1111/jep.13118

[jlcd70116-bib-0025] Lazzarini, P. A. , J. Geraghty , E. M. Kinnear , M. Butterworth , and D. Ward . 2013. “Research Capacity and Culture in Podiatry: Early Observations Within Queensland Health.” Journal of Foot and Ankle Research 6: 1. 23302627 10.1186/1757-1146-6-1 PMC3549934

[jlcd70116-bib-0026] Lee, S. A. , K. Byth , J. A. Gifford , et al. 2020. “Assessment of Health Research Capacity in Western Sydney Local Health District (WSLHD): A Study on Medical, Nursing and Allied Health Professionals.” Journal of Multidisciplinary Healthcare 13: 153–163. 32103975 10.2147/JMDH.S222987 PMC7024741

[jlcd70116-bib-0027] Lizarondo, L. , K. Grimmer‐Somers , and S. Kumar . 2011. “A Systematic Review of the Individual Determinants of Research Evidence Use in Allied Health.” Journal of Multidisciplinary Healthcare 4: 261–272. 21847348 10.2147/JMDH.S23144 PMC3155856

[jlcd70116-bib-0028] Luckson, M. , F. Duncan , A. Rajai , and C. Haigh . 2018. “Exploring the Research Culture of Nurses and Allied Health Professionals (AHP s) in a Research‐Focused and a Non‐Research‐Focused Healthcare Organisation in the UK.” Journal of Clinical Nursing 27: e1462–e1476. 29322683 10.1111/jocn.14264

[jlcd70116-bib-0029] Michie, S. , L. Atkins , and R. West . 2014. A Guide to Designing Interventions, 1003–1010. 1st ed. Silverback Publishing.

[jlcd70116-bib-0030] Newington, L. , M. Wells , A. Adonis , et al. 2021. “A Qualitative Systematic Review and Thematic Synthesis Exploring the Impacts of Clinical Academic Activity by Healthcare Professionals Outside Medicine.” BMC Health Services Research 21: 400. 33926441 10.1186/s12913-021-06354-y PMC8082861

[jlcd70116-bib-0031] NHS England . 2019. “The NHS Long Term Plan.” NHS England.

[jlcd70116-bib-0032] Pager, S. , L. Holden , and X. Golenko . 2012. “Motivators, Enablers, and Barriers to Building Allied Health Research Capacity.” Journal of Multidisciplinary Healthcare 5: 53–59. 22396626 10.2147/JMDH.S27638 PMC3292402

[jlcd70116-bib-0033] Royal College of Speech and Language Therapy . 2022a. “Research Practitioner Framework Resource Map.” https://www.rcslt.org/members/research/careers/slt‐research‐practitioner‐framework‐resource‐map/.

[jlcd70116-bib-0034] Royal College of Speech and Language Therapy . 2022b. “The RCSLT Strategic Vision: 2022–2027.” RCSLT‐Strategic‐Vision‐2022‐2027.pdf.

[jlcd70116-bib-0035] Sharma, A. , N. T. Minh Duc , T. Luu Lam Thang , et al. 2021. “A Consensus‐Based Checklist for Reporting of Survey Studies (CROSS).” Journal of General Internal Medicine 36: 3179–3187. 33886027 10.1007/s11606-021-06737-1 PMC8481359

[jlcd70116-bib-0037] Wagner, A. K. , J. Mcelligott , E. P. Wagner , and L. H. Gerber . 2005. “Measuring Rehabilitation Research Capacity: Report From the AAPM&R Research Advisory Committee.” American Journal of Physical Medicine & Rehabilitation 84: 955–968. 16327412 10.1097/01.phm.0000187860.11221.8c

[jlcd70116-bib-0038] Wenke, R. , and S. Mickan . 2016. “The Role and Impact of Research Positions Within Health Care Settings in Allied Health: A Systematic Review.” BMC Health Services Research 16: 1–10. 27495229 10.1186/s12913-016-1606-0 PMC4974741

[jlcd70116-bib-0039] Wenke, R. , C. Noble , K. A. Weir , and S. Mickan . 2020. “What Influences Allied Health Clinician Participation in Research in the Public Hospital Setting: A Qualitative Theory‐Informed Approach.” BMJ Open 10, no. 8: e036183. 10.1136/bmjopen-2019-036183 PMC7443264 32819986

[jlcd70116-bib-0040] Westwood, G. , A. Richardson , S. Latter , J. Macleod Clark , and M. Fader . 2018. “Building Clinical Academic Leadership Capacity: Sustainability Through Partnership.” Journal of Research in Nursing 23: 346–357. 34394442 10.1177/1744987117748348 PMC7932196

[jlcd70116-bib-0041] Whitworth, A. , S. Haining , and H. Stringer . 2012. “Enhancing Research Capacity Across Healthcare and Higher Education Sectors: Development and Evaluation of an Integrated Model.” BMC Health Services Research 12: 1–10. 22929175 10.1186/1472-6963-12-287 PMC3471044

