# Supplementary material for: Use of the Capability, Opportunity, Motivation‐Behaviour Model and Theoretical Domains Framework to Understand Barriers and Enablers of Research Capacity and Culture for Speech and Language Therapy Staff
Source: Int J Lang Commun Disord. 2025 Sep 10;60(5):e70116. doi: 10.1111/1460-6984.70116 (PMC12421707; doi:10.1111/1460-6984.70116)
Supplement: Supplementary file 2 — Research Capacity and Culture in Speech and Language Therapy Staff Survey. [file JLCD-60-0-s004.pdf]

Supplementary File 2.

Research Capacity and Culture in Speech and Language Therapy Staff Survey.

Demographic Information

What is your current service area/area of clinical specialism? *If more than one, select the two which you currently spend most time working into.*

Adult Learning Disability – Community
Adult Learning Disability – Criminal Justice
Adult Learning Disability – Inpatient
Adult Neurorehabilitation/Neuropsychiatry
Adult Psychiatry Services - Community
Adult Psychiatry Services – Criminal Justice
Adult Psychiatry Services – Inpatient
Augmentative and Alternative Communication
Dysphagia
Neurodiversity
Paediatric Learning Disability – Community
Paediatric Learning disability – Inpatient
Paediatric Mental Health – Community
Paediatric Mental Health – Inpatient
Voice

How long have you been qualified as a Speech and Language Therapist? (If applicable)

- 0-5 years
- 6-10 years
- 11-15 years
- 16-20 years
- 20 years +
- Not Applicable

Please indicate your highest level of academic or professional qualification.

- No formal qualification
- Certificate/Diploma
- Degree
- Masters /Post Graduate
- Doctorate
- Other

If you have completed a degree (undergraduate/postgraduate), did you conduct a research study as part of this?

- Yes
- No
- Don’t know
- Not Applicable

Are you enrolled in any Research Related Study?

- Yes
- No

If yes, please indicate What Level of Study You Are Enrolled In

- In Work Training
- Undergraduate
- Postgraduate Certificate/Diploma
- Postgraduate master's Level
- Doctoral Level Research
- Post Doctoral Study
- Not Applicable

Is research engagement/activity discussed as part of your annual appraisal?

- Yes routinely.
- Only if I am currently involved in research or if I ask to discuss it.
- No

Are research related activities included as part of your job role?

- Yes
- No
- Don't Know

How much of your time in your current role is formally allocated for research or research-related activity?

- Less than 25% of my time
- More than 25 of my time but less than 50% of my time
- More than 50% of my time but less than 75% of my time
- More than 75% of my time.

How would you describe your status based on the following descriptions?

- Research Conscious (e.g. An awareness of research in the workplace and the skills to seek, critique and use evidence already in the public domain as part of daily practice).
- Research Participative (e.g. Involved as a member of a research team or project, Signposting patient participants to research projects, may be involved in carrying out novel complex interventions or developing research ideas and projects with academics).
- Research Active (e.g. Undertaking a research degree at post graduate level or have research embedded in their substantive job with numerous links to academics and research orientated information and support).
- None of the above.

Capability - Psychological Capability

Please select the research activity you feel you have the knowledge to conduct. (Yes/No/Don't know)

- Searching the literature
- Critically appraising the literature
- Using a computer referencing system
- Designing an audit
- Designing a service evaluation.
- Registering a service evaluation or audit with the NHS trust.
- Writing a research proposal

- Submitting an ethics application.
- Designing a way to collect quantitative data e.g. survey, observation.
- Designing a way to collect qualitative data e.g. focus group/interview.
- Using computer data management systems to analyse quantitative data e.g. SPSS
- Using computer data management systems to analyse qualitative data e.g. NVIVO.
- Writing a research report.
- Writing for publication in peer reviewed journal.
- Writing to share research information with stakeholders. Service users, family members etc.
- Providing advice to others about research related activities.
- Gaining management support for research /development activity.

Please select the research activity you feel you have the skills to conduct (Yes/no. don't know)

- Searching the literature
- Critically appraising the literature
- Using a computer referencing system
- Designing an audit
- Designing a service evaluation.
- Registering a service evaluation or audit with the NHS trust.
- Writing a research proposal
- Submitting an ethics application.
- Designing a way to collect quantitative data e.g. survey, observation.
- Designing a way to collect qualitative data e.g. focus group/interview.
- Using computer data management systems to analyse quantitative data e.g. SPSS
- Using computer data management systems to analyse qualitative data e.g. NVIVO.
- Writing a research report.
- Writing for publication in peer reviewed journal.
- Writing to share research information with stakeholders. Service users, family members etc.
- Providing advice to others about research related activities.
- Gaining management support for research /development activity.

Opportunity – Physical

Thinking about your role and your opportunity to engage in research. Do you have the following (Yes/no/don't know).

- Allocated time to complete research training/activities.
- Allocated time to be involved in research?
- Required Equipment available e.g. audio-visual recording equipment, assessments, survey software.
- Admin support for research.
- Access to Software or statistical packages for research.
- Library access.
- Funding within NHS trust to support SLT staff research.
- Appropriate Research supervision.
- Support to access (funding/time) research training within the NHS trust.
- Support (Funding/time) to access research training external to the NHS trust.
- Support (time/funding) for SLT staff to attend research conferences.
- Opportunities for SLT staff to be involved in delivering research.
- Opportunities for SLT staff to be a principal investigator.
- Joint university/NHS trust contracts for SLT clinical academics.

Opportunity – Social

Within the setting/team you work into, do you have the following? (Yes/no/don't know)

Information about ongoing research projects within the NHS trust.

- Information about how the NHS trust supports/promotes clinical academic careers.
- Support for research activity from colleagues.
- Support for research activity from management.
- Colleagues in your setting taking part in research activity.
- Encouragement/support to apply for external research funding.
- Encouragement/support to apply for internal research funding.
- Formal research supervision/mentorship available for SLT staff (e.g. via academic links?)
- Informal research supervision/ mentorship for SLT staff.

Motivation – Reflexive

Please answer the following selecting Yes/No/Don't Know.

- Do you see research activity as part of your job role?
- Do you see research activity as part of your professional identity?
- Would you like to increase the amount of research related activities you take part in?
- Do you think clinical practice improves if clinicians take part in research activity?
- Do you think job satisfaction improves if clinicians take part in research activity?

Please answer the following selecting Yes/No/Don't Know.

- Are you motivated by the idea of conducting research into your clinical specialism?

Motivation – Automatic

Free text question

- How does thinking about completing research activities make you feel?
