# Supplementary material for: Use of the Capability, Opportunity, Motivation‐Behaviour Model and Theoretical Domains Framework to Understand Barriers and Enablers of Research Capacity and Culture for Speech and Language Therapy Staff
Source: Int J Lang Commun Disord. 2025 Sep 10;60(5):e70116. doi: 10.1111/1460-6984.70116 (PMC12421707; doi:10.1111/1460-6984.70116)
Supplement: Supplementary file 3 — Barriers and Enablers Data from Participant Questionnaire. [file JLCD-60-0-s002.pdf]

Supplementary File 3. Barriers and Enablers Data from Participant Questionnaire.

Com-b Domain	TDF Domain	Subcategory	Barrier n (%)	Enabler n (%)	Don't know n (%)
		**Individual Level**			
Capability - Psychological	Knowledge	**Knowledge to Engage in Evidence Based Practice.**			
		Searching the literature.	4 (7.02%) Weak	52 (91.23%) Strong	1 (1.75%)
		Critically appraising the literature.	10 (17.54%) Weak	44 (77.19%) Strong	3 (5.26%)
		Using a computer referencing system.	17 (29.82%) Weak	35 (61.40%) Moderate	5 (8.77%)
		**Knowledge to Gather and Use Feedback and Information from Service Users to Evaluate Care.**			
		Designing a way to collect quantitative data e.g survey, observation.	12 (21.05%) Weak	38 (66.67%) Strong	7 (12.28%)
		Designing a way to collect qualitative data e.g. focus group/interview.	10 (17.54%) Weak	44 (77.19%) Strong	3 (5.26%)
		**Knowledge to Monitoring and Systematically Evaluating Clinical Practice.**			
		Designing an audit.	22 (38.60%) Moderate	31 (54.39%) Moderate	4 (7.02%)
		Designing a service evaluation.	13 (22.81%) Weak	39 (68.42%) Strong	5 (8.77%)
		Registering a service evaluation or audit with your health trust.	33 (57.89%) Moderate	17 (29.82%) Weak	7 (12.28%)
		**Knowledge for More Advanced Research Activities.**			
		Writing a research proposal	33 (57.89%) Moderate	19 (33.33%) Weak	5 (8.77%)
		Submitting an ethics application.	41 (71.93%)	9 (15.79%)	7 (12.28%)

Com-b Domain	TDF Domain	Subcategory	Barrier n (%)	Enabler n (%)	Don't know n (%)
			Strong	Weak	
		Using computer data management systems to analyse quantitative data e.g. SPSS.	41 (71.93%) Strong	12 (21.05%) Weak	4 (7.02%)
		Using computer data management systems to analyse qualitative data e.g. NVIVO.	48 (84.21%) Strong	5 (8.77%) Weak	4 (7.02%)
		Writing a research report.	27 (47.37%) Moderate	24 (42.11%) Moderate	6 (10.53%)
		Writing for publication in a peer reviewed journal.	42 (73.68%) Strong	10 (17.54%) Weak	5 (8.77%)
		Writing to share research information with stakeholders. Service users, family members etc.	29 (50.88%) Moderate	21 (36.84%) Moderate	7 (12.28%)
		Providing advice to others about research related activities.	42 (73.68%) Strong	9 (15.79%) Weak	6 (10.53%)
		Gaining management support for research /development activity.	22 (38.60%) Moderate	23 (40.35%) Moderate	12 (21.05%)
Capability - Psychological	Cognitive and interpersonal skills	**Skills to Engage in Evidence Based Practice.**			
		Searching the literature.	6 (10.53%) Weak	49 (85.96%) Strong	2 (3.51%)
		Critically appraising the literature.	9 (15.79%) Weak	42 (73.68%) Strong	6 (10.53%)
		Using a computer referencing system.	19 (33.33%) Weak	34 (59.65%) Moderate	4 (7.02%)
		**Skills to Gather and Use Feedback and Information from Service Users to Evaluate Care.**			
		Designing a way to collect quantitative data e.g. survey, observation.	21 (36.84%) Moderate	32 (56.14%) Moderate	4 (7.02%)
		Designing a way to collect qualitative data e.g. focus group/interview.	14 (24.56%) Weak	38 (66.67%) Strong	5 (8.77%)
		**Skills to Monitoring and Systematically Evaluating Clinical Practice.**			

Com-b Domain	TDF Domain	Subcategory	Barrier n (%)	Enabler n (%)	Don't know n (%)
		Designing an audit.	25 (43.86%) Moderate	28 (49.12%) Moderate	4 (7.02%)
		Designing a service evaluation.	19 (33.33%) Weak	33 (57.89%) Moderate	5 (8.77%)
		Registering a service evaluation or audit with your NHS trust.	29 (50.88%) Moderate	24 (42.11%) Moderate	4 (7.02%)
		**Skills for More Advanced Research Activities.**			
		Writing a research proposal	28 (49.12%) Moderate	21 (36.84%) Moderate	8 (14.04%)
		Submitting an ethics application.	38 (66.67%) Strong	13 (22.81%) Weak	6 (10.53%)
		Using computer data management systems to analyse quantitative data e.g. SPSS	47 (82.46%) Strong	5 (8.77%) Weak	5 (8.77%)
		Using computer data management systems to analyse qualitative data e.g. NVIVO	43 (75.44%) Strong	8 (14.04%) Weak	6 (10.53%)
		Writing a research report.	25 (43.86%) Moderate	25 (43.86%) Moderate	7 (12.28%)
		Writing for publication in a peer reviewed journal	37 (64.91%) Moderate	13 (22.81%) Weak	7 (12.28%)
		Writing to share research information with stakeholders. Service users, family members etc.	28 (49.12%) Moderate	23 (40.35%) Moderate	6 (10.53%)
		Providing advice to others about research related activities.	36 (63.16%) Strong	12 (21.05%) Weak	9 (15.79%)
		Gaining management support for research/development activity.	23 (40.35%) Moderate	24 (42.11%) Moderate	10 (17.54%)
Motivation - Reflexive	Professional/ Social role and identity	Do you see research activity as part of your job role?	11 (19.30%) Weak	38 (66.67%) Strong	8 (14.04%)
		Do you see research activity as part of your professional identity?	15 (26.32%) Weak	38 (66.67%) Strong	4 (7.02%)

Com-b Domain	TDF Domain	Subcategory	Barrier n (%)	Enabler n (%)	Don't know n (%)
Motivation - Reflexive	Intentions	Would you like to increase the amount of research related activities you take part in?	7 (12.28%) Weak	41 (71.93%) Strong	9 (15.79%)
Motivation - Reflexive	Beliefs about consequences	Do you think clinical practice improves if clinicians take part in research activity?	0 (0.00%) Weak	52 (91.23%) Strong	5 (8.77%)
		Do you think job satisfaction improves if clinicians take part in research activity?	6 (10.53%) Weak	32 (56.14%) Moderate	19 (33.33%)
Motivation - Automatic	Emotion	Are you motivated by the idea of conducting research into your clinical specialism?	7 (12.28%) Weak	41 (71.93%) Strong	9 (15.79%)
		Respondents' enabler and barrier emotions when thinking about research activity.	18 (31.58%) Weak	15 (26.32%) Weak	Both emotions 24 (42.11%) Moderate

Team and Organisational levels

Com-b Domain	TDF Domain	Subcategory	Barrier n (%)	Enabler n (%)	Don't know n (%)
Opportunity - Physical	Environmental Context and Resources	**Time.**			
		Allocated time to be involved in research.	37 (64.91%) Moderate	13 (22.81%) Weak	7 (12.28%)
		**Time for Opportunities for development and mentoring.**			
		Allocated time to complete research training/activities.	28 (49.12%) Moderate	24 (42.11%) Moderate	5 (8.77%)
		Support to access (funding/time) research training within your NHS trust.	18 (31.58%) Weak	13 (22.81%) Weak	26 (45.61%)
		Support (Funding/time) to access research training provided by external providers.	18 (31.58%) Weak	12 (21.05%) Weak	27 (47.37%)

Com-b Domain	TDF Domain	Subcategory	Barrier n (%)	Enabler n (%)	Don’t know n (%)
		Appropriate Research supervision.	22 (38.60%) Moderate	9 (15.79%) Weak	26 (45.61%)
		Formal research supervision/mentorship available for SALT staff (eg via academic links?)	20 (35.09%) Moderate	7 (12.28%) Weak	30 (52.63%)
		Informal research supervision/ mentorship for SLT staff.	14 (24.56%) Weak	16 (28.07%) Weak	27 (47.37%)
		Support (time/funding) for SLT staff to attend research conferences.	14 (24.56%) Weak	28 (49.12%) Moderate	15 (26.32%)
		**Resources and Infrastructure.**			
		Library access.	0 (0.00%) None	56 (98.25%) Strong	1 (1.75%)
		Required Equipment available e.g., audio-visual recording equipment, assessments, survey software.	32 (56.14%) Moderate	12 (21.05%) Weak	13 (22.81%)
		Admin support for research.	35 (61.40%) Moderate	5 (8.77%) Weak	17 (29.82%)
		Access to Software or statistical packages for research.	36 (63.16%) Moderate	4 (7.02%) Weak	17 (29.82%)
		**Funding and Job Opportunities.**			
		Funding within your NHS trust to support SLT staff research.	15 (26.32%) Weak	13 (22.81%) Weak	29 (50.88%)
		Opportunities for SLT staff to be involved in delivering research.	13 (22.81%) Weak	25 (43.86%) Moderate	19 (33.33%)
		Opportunities for SLT staff to be a principal investigator.	20 (35.09%) Moderate	11 (19.30%) Weak	26 (45.61%)
		Joint university/trust contracts for SLT clinical academics.	21 (36.84%) Moderate	9 (15.79%) Weak	27 (47.37%)
Opportunity - Social	Social influences	**Information Provided.**			
		Information about ongoing research projects within the Trust.	19 (33.33%) Weak	27 (47.37%) Moderate	11 (19.30%)

Com-b Domain	TDF Domain	Subcategory	Barrier n (%)	Enabler n (%)	Don’t know n (%)
		Information about how the Trust supports/promotes clinical academic careers.	23 (40.35%) Moderate	12 (21.05%) Weak	22 (38.60%)
		**Social Support.**			
		Support for research activity from colleagues.	10 (17.54%) Weak	32 (56.14%) Moderate	15 (26.32%)
		Support for research activity from management.	12 (21.05%) Weak	26 (45.61%) Moderate	19 (33.33%)
		Colleagues in your setting taking part in research activity.	15 (26.32%) Weak	33 (57.89%) Moderate	9 (15.79%)
		Encouragement/support to apply for external research funding.	18 (31.58%) Weak	13 (22.81%) Weak	26 (45.61%)
		Encouragement/support to apply for internal research funding.	17 (29.82%) Weak	15 (26.32%) Weak	25 (43.86%)
