# Supplementary figures and images for: Use of the Capability, Opportunity, Motivation‐Behaviour Model and Theoretical Domains Framework to Understand Barriers and Enablers of Research Capacity and Culture for Speech and Language Therapy Staff
Source: Int J Lang Commun Disord. 2025 Sep 10;60(5):e70116. doi: 10.1111/1460-6984.70116 (PMC12421707; doi:10.1111/1460-6984.70116)

## Supplementary File 4. Respondents' Emotions When Thinking About Research

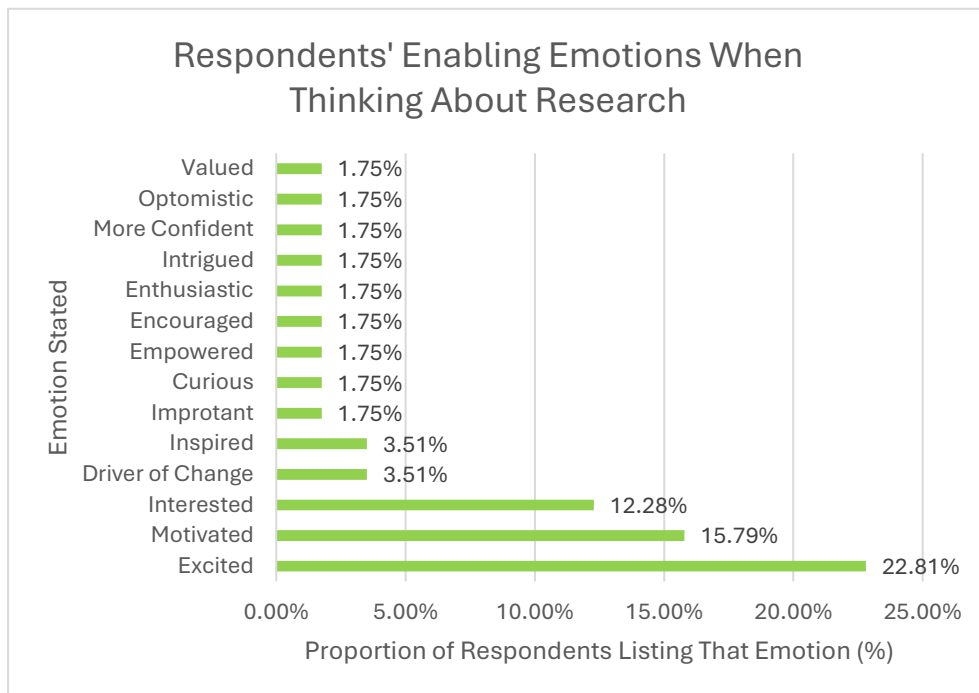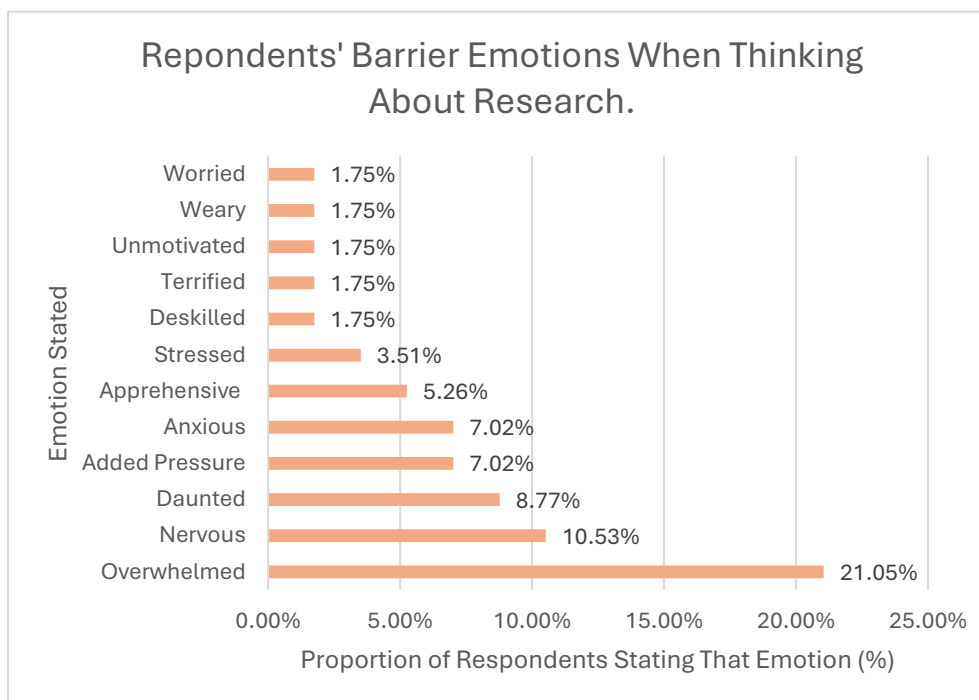

Supplement: Supplementary file 4 — Respondents’ Emotions When Thinking About Research [file JLCD-60-0-s003.pdf]
